# Cell-free nuclear, but not mitochondrial, DNA concentrations correlate with the early host inflammatory response after severe trauma

**DOI:** 10.1038/s41598-019-50044-z

**Published:** 2019-09-20

**Authors:** Julie A. Stortz, Russell B. Hawkins, David C. Holden, Steven L. Raymond, Zhongkai Wang, Scott C. Brakenridge, Joseph Cuschieri, Frederick A. Moore, Ronald V. Maier, Lyle L. Moldawer, Philip A. Efron

**Affiliations:** 1 Department of Surgery, University of Florida College of Medicine, Gainesville, FL 32610 USA; 2 Department of Biostatistics, University of Florida College of Medicine, Gainesville, FL 32610 USA; 3 Department of Surgery, University of Washington School of Medicine, Seattle, WA 98104 USA

**Keywords:** Mechanisms of disease, Diagnostic markers

## Abstract

Severe blunt trauma is associated with an early ‘*genomic storm*’ which causes simultaneous up- and down-regulation of host protective immunity. Excessive inflammation can lead to organ injury. In the absence of infection, the inflammatory response is presumably driven by release of endogenous alarmins called danger-associated molecular patterns (DAMPs), which initiate immune responses through pattern-recognition receptors (PRR). Here we examined the relationship between concentrations of cell-free (cf) nuclear DNA (ncDNA) and mitochondrial DNA (mtDNA) within 24 hours post trauma with circulating leukocyte transcriptomics and plasma IL-6 concentrations, as well as the patients’ clinical trajectories. In 104 patients enrolled from two level-1 trauma centers, ncDNA and mtDNA concentrations were increased within 24 hours of severe trauma, but only ncDNA concentrations correlated with leukocyte gene expression and outcomes. Surprisingly, ncDNA, not mtDNA concentrations, were significantly elevated in trauma patients who developed chronic critical illness versus rapid clinical recovery. Plasma IL-6 and leukocyte transcriptomics were better predictors of outcomes than cfDNA levels. Although mtDNA and ncDNA are significantly increased in the immediate post-trauma period, the dramatic inflammatory and gene expression changes seen after severe trauma are only weakly correlated with ncDNA concentrations, and more importantly, mtDNA concentrations are not associated with adverse clinical trajectories.

## Introduction

Severe blunt trauma remains a common morbid event throughout the world and is associated with what has been termed an early *‘genomic’* or *‘cytokine’* storm^1,2^. In the absence of any obvious microbial infection, this marked inflammatory response is thought to arise from the release of endogenous alarmins, or danger-associated molecular patterns (DAMPs)^3,4^. The list of known endogenous alarmins is expansive and includes both proteins and nucleic acids. Many DAMPs, including cell-free DNA (cfDNA), which is comprised of either nuclear DNA (ncDNA) or mitochondrial DNA (mtDNA), are released in degraded and oxidized forms following traumatic injury as a consequence of cellular death, or in some cases, active secretion^4,5^. These endogenous alarmins often act through the same pattern recognition receptors (PRRs) and signaling pathways that are used by the host to recognize microbial products, and this gives strength to the observation that the early inflammatory response to severe blunt trauma and microbial infection have similarities^4,6^. Unlike ncDNA, mtDNA is circular, has a higher G-C content, and has different methylation patterns^7^. In addition, mtDNA does not circulate tightly bound to nucleosomes, arguing that it should be preferentially recognized by both binding proteins and PRRs^7,8^.

Previous research has demonstrated variable association between mtDNA concentrations and trauma severity, post-injury complications and mortality^9–14^. Similarly, additional alarmins, including HMGB1, ATP, cytochrome C, TFAM, and hyaluronan, have been associated with outcomes in severe trauma^15–17^. However, few if any studies have prospectively examined a link between cfDNA concentrations, the host immunological response, and clinical outcomes after severe blunt trauma. In fact, in a recent review, Lubkin *et al*. concluded that further research is required to determine if cfDNA, and specifically mtDNA, induces an inflammatory response in the host or merely is a marker of cellular injury^18^.

Here, we test the hypothesis that circulating cfDNA, particularly mtDNA concentrations, are associated with the exaggerated inflammatory response that leads to organ injury and adverse clinical outcomes. We simultaneously examined representative cell-free ncDNA and mtDNA concentrations, blood leukocyte transcriptomics, plasma IL-6 concentrations and clinical outcomes in subjects with severe blunt trauma without traumatic brain injury. We dichotomized clinical trajectories based on organ injury and duration of intensive care stay since this trauma population exhibits a low inpatient mortality, but is heterogeneous with respect to outcomes, demonstrating significant variation in time to recovery^2,19,20^.

## Results

### Patient demographics and outcomes

A total of 104 subjects were enrolled in the trauma cohort. Forty-three patients were enrolled at UF Health Shands Hospital, University of Florida, and 61 patients at Harborview Medical Center, University of Washington. The overall cohort was predominantly comprised of white (86%) males (72%) with a median age of 48 years (Table 1). Of the 104 trauma subjects enrolled, three patients experienced early death (3%), 22 met the criteria for experiencing chronic critical illness (CCI) (21%), and the remainder demonstrated rapid recovery (RAP). Twenty-eight day mortality for the cohort was 6%. Not unexpectedly, those that developed CCI were generally older (56 [44, 65] years versus 46 [26, 59] years, p = 0.016; medians, [quartiles]), had greater APACHE II (Acute Physiology and Chronic Health Evaluation) scores (29 [23, 36] versus 22 [16, 27], p = 0.003), and required more blood transfusions (7.0 [4.7, 10.5] versus 2.3 [1.0, 7.0] U PRBC, p = 0.002) and crystalloid resuscitation (11.0 [7.4, 15.3] versus 8.4 [6.4, 11.4] L, p < 0.02). Surprisingly, injury severity scores (ISS) did not significantly differ among groups.

**Table 1:** Baseline and Injury Characteristics of the Study Subjects.

	Total Cohort (n = 104)	RAP (76.0%) (n = 79)	CCI (21.1%) (n = 22)	Early Deaths (2.9%) (n = 3)	P-value (RAP vs CCI)
Age	48 (28, 60)	46 (26, 59)	56 (44, 65)	50 (38, 73)	0.0163
Male sex, n (%)	75 (72.1)	57 (72.2)	18 (81.8)	0 (0)	0.4218
Race, n (%)					0.0961
White	90 (86.5)	68 (86.1)	19 (86.4)	3 (100)	
African American	8 (7.7)	8 (10.5)	0 (0)	0 (0)	
American Indian	2 (1.9)	1 (1.3)	1 (4.5)	0 (0)	
Pacific Islander	0 (0)	0 (0)	0 (0)	0 (0)	
Asian	3 (2.9)	2 (2.5)	1 (4.5)	0 (0)	
Unknown	75 (72.1)	57 (72.2)	18 (81.8)	0 (0)	
Ethnicity (non-hispanic), n (%)	98 (94.2)	75 (94.9)	20 (90.9)	3 (100)	0.4546
BMI	27.2 (24.7, 32.3)	26.9 (24.5, 32.4)	27.6 (25.5, 32.3)	30.5 (29.1, 37)	0.4057
Number of comorbidities, n (%)					0.5662
0	35 (33.7)	28 (35.4)	7 (31.8)	0 (0)	
1	37 (35.6)	29 (36.7)	6 (27.3)	2 (66.7)	
≥2	32 (30.8)	22 (27.8)	9 (40.9)	1 (33.3)	
APACHE II score	23 (17, 29)	22 (16, 27)	29 (23, 36)	37 (30, 41)	0.0031
GCS	7.5 (3, 10)	8 (3, 11)	6 (3, 10)	3 (3, 3)	
ISS	34 (24, 41)	29 (22, 41)	34 (27, 43)	42 (41, 43)	0.1764
Injury mechanism, n (%)					0.8748
Fall	8 (7.7)	7 (8.9)	1 (4.5)	0 (0)	
Motor vehicle collision	90 (86.5)	67 (84.8)	20 (90.9)	3 (100)	
Other	6 (5.8)	5 (6.3)	1 (4.5)	0 (0)	
Total transfusion (ml) within 24 hours					
PRBC (units)	4.5 (1.4, 8.2)	2.3 (1, 7)	7 (4.7, 10.5)	4.7 (4.3, 116.2)	0.0020
FFP (units)	1.4 (0, 3.7)	1.3 (0, 2.9)	2.9 (0, 4.8)	2.8 (1, 73.3)	0.0548
Total crystalloid (ml) within 24 hours	8717.5 (6784, 12257)	8446 (6383, 11425)	11081.5 (7376, 15259)	10123 (8318, 13039)	0.0195
Worst base deficit (meq/L) within 24 hours	−8.3 (−11.8, −4)	−7.2 (−10.7, −3.4)	−11.8 (−13.8, −7)	−16 (−17.4, −8.5)	0.0078
Highest lactate (mmol/L) within 24 hours	4.8 (3.3, 6.6)	4.4 (3.2, 5.7)	6.3 (3.5, 7.8)	7.9 (2.6, 11)	0.0189
Lowest ED SBP (mm Hg)	85 (68.5, 97)	88 (74, 100)	68 (56, 89)	69 (49, 92)	0.0033
Initial ED SBP (mm Hg)	115 (98, 134)	119 (104, 137)	105.5 (96, 117)	92 (57, 127)	0.0647
ED systolic <90 mm Hg, n (%)	19 (18.3)	14 (17.7)	3 (13.6)	2 (66.7)	0.7586

*Abbreviations*: RAP = rapid recovery; CCI = chronic critical illness; BMI = body mass index; APACHE II = acute physiology and chronic health evaluation II; GCS = Glasgow Coma Score; ISS = injury severity score; PRBC = packed red blood cells; FFP = fresh frozen plasma; ED = emergency department; SBP = systolic blood pressure. Data are represented as median (25^th^, 75^th^) if not indicated.

Overall, the total length of hospital and ICU stays for all patients were 18 [11, 29] and 8 [4, 15] days, respectively (Table 2). As a whole, average ‘time to recovery’ (TTR) was 8.5 [4.5, 29] days. Over one third of subjects (36%) had noninfectious complications, while 42% had nosocomial infections. The primary infectious complication was pneumonia (21%) followed by surgical site infection (13%). Forty percent were discharged home, either with or without home healthcare.

**Table 2:** Patient Outcomes and Complications.

	Total Cohort (n = 104)	RAP (76.0%) (n = 79)	CCI (21.1%) (n = 22)	Early Deaths (2.9%) (n = 3)	P-value (RAP vs CCI)
28 day-mortality, n (%)	6 (5.8)	0 (0)	3 (13.6)	3 (100)	0.0092
Hospital length of stay (days)	18 (11.5, 29.5)	17 (11, 24)	39 (26, 47)	3 (2, 5)	<0.0001
ICU length of stay (days)	8 (4, 15)	6 (3, 11)	24 (21, 32)	2 (2, 5)	<0.0001
ICU-free days^a^ (out of 28-day)	19.5 (10, 24)	22 (17, 25)	1.5 (0, 5)	0 (0, 0)	<0.0001
Mechanical ventilation, n (%)	90 (86.5)	65 (82.3)	22 (100)	3 (100)	0.0359
Ventilator-free days^b^ (out of 28-day)	24 (17, 27)	26 (23, 27)	9.5 (0, 13)	0 (0, 0)	<0.0001
Multiple organ failure, n (%)	16 (15.4)	5 (6.3)	10 (45.5)	1 (33.3)	<0.0001
Maximum Denver MOF score	1 (0, 2)	0 (0, 1)	2 (2, 5)	0 (0, 5)	<0.0001
Time to recovery^c^ (days)	8.5 (4.5, 29)	7 (3, 12)	26 (19, 29)	30 (30, 30)	<0.0001
Cardio recovery	1 (1, 2)	1 (1, 2)	11 (2, 26)	2 (1, 29)	<0.0001
Hemo recovery	4 (1, 6)	4 (1, 6)	5.5 (2, 13)	2 (1, 5)	0.041
Hepatic recovery	1 (1, 2)	1 (1, 2)	2 (1, 8)	2 (1, 4)	0.0028
Renal recovery	1 (1, 2)	1 (1, 2)	5.5 (1, 25)	2 (1, 29)	<0.0001
Respiratory recovery	3 (1, 11)	2 (1, 5)	19 (15, 25)	29 (2, 29)	<0.0001
Noninfectious complications^d^, n (%)	37 (35.6)	19 (24.1)	16 (72.7)	2 (66.7)	<0.0001
Infectious complications, n (%)	44 (42.3)	25 (31.6)	19 (86.4)	0 (0)	<0.0001
Infection source, n (%)					
Pneumonia	22 (21.2)	11 (13.9)	11 (50)	0 (0)	0.0008
Pseudomembranous colitis	10 (9.6)	6 (7.6)	4 (18.2)	0 (0)	0.2183
UTI	8 (7.7)	4 (5.1)	4 (18.2)	0 (0)	0.0659
Surgical site infection	13 (12.5)	6 (7.6)	7 (31.8)	0 (0)	0.0010
Blood stream infection	2 (1.9)	0 (0)	2 (9.1)	0 (0)	0.0457
Empyema	1 (0.9)	0 (0)	1 (4.5)	0 (0)	0.2178
Other	5 (4.8)	1 (1.3)	4 (18.2)	0 (0)	0.0076
Time to first nosocomial infection (days)	5.5 (3, 8.5)	4 (2, 11)	6.5 (4.5, 8)	NA	0.5371
Number of nosocomial infections per patient, n (%)					<0.0001
0	68 (65.4)	59 (74.7)	6 (27.3)	3 (100)	
1	26 (25)	18 (22.8)	8 (36.4)	0 (0)	
≥2	10 (9.6)	2 (2.5)	8 (36.4)	0 (0)	
Discharge disposition, n (%)					0.0030
“Good” disposition					
Home	27 (26)	25 (31.6)	2 (9.1)	0 (0)	
Home with home health care services	15 (14.4)	12 (15.2)	3 (13.6)	0 (0)	
Inpatient rehabilitation facility	21 (20.2)	16 (20.3)	5 (22.7)	0 (0)	
“Poor” disposition					
Skilled nursing facility	30 (28.8)	24 (30.4)	6 (27.3)	0 (0)	
Long term acute care facility	4 (3.8)	2 (2.5)	2 (9.1)	0 (0)	
In-hospital death	7 (6.7)	0 (0)	4 (18.2)	3 (100)	

*Abbreviations*: RAP = rapid recovery; CCI = chronic critical illness; SOFA = sequential organ failure assessment; GG = glue grant; ARDS = acute respiratory distress syndrome; DVT = deep venous thrombosis; PE = pulmonary embolism; UTI = urinary tract infection; SD = standard deviation.

Data are represented as median (25^th^, 75^th^) if not indicated.

^a^The number of ICU-free days was calculated as 28 minus the number of days or part-days in the ICU. Patients who died within 28 days were assigned zero ICU-free days.

^b^The number of ventilator-free days was calculated as 28 minus the number of days or part-days on mechanical ventilation. Patients who died within 28 days were assigned zero ventilator-free days.

^c^Time to recovery is defined in Supplemental Table S1.

^d^Non-infectious complications included pathologies such as acute respiratory distress syndrome, deep venous thrombosis, pulmonary embolism, myocardial infarction, among others.

As expected, patients who developed CCI had a significantly higher incidence of both infectious (86%) and noninfectious (73%) complications compared to patients who rapidly recovered (32% and 24%, respectively; p < 0.001). Finally, while nearly 70% of RAP patients were discharged to home or to rehabilitation facilities, more than half of patients who developed CCI died or were discharged to facilities associated with poor long-term outcomes (i.e. skilled nursing facilities, long-term acute care) (Table 2).

### Plasma cell-free DNA (cfDNA) and transcriptomic responses

**Figure 1:**
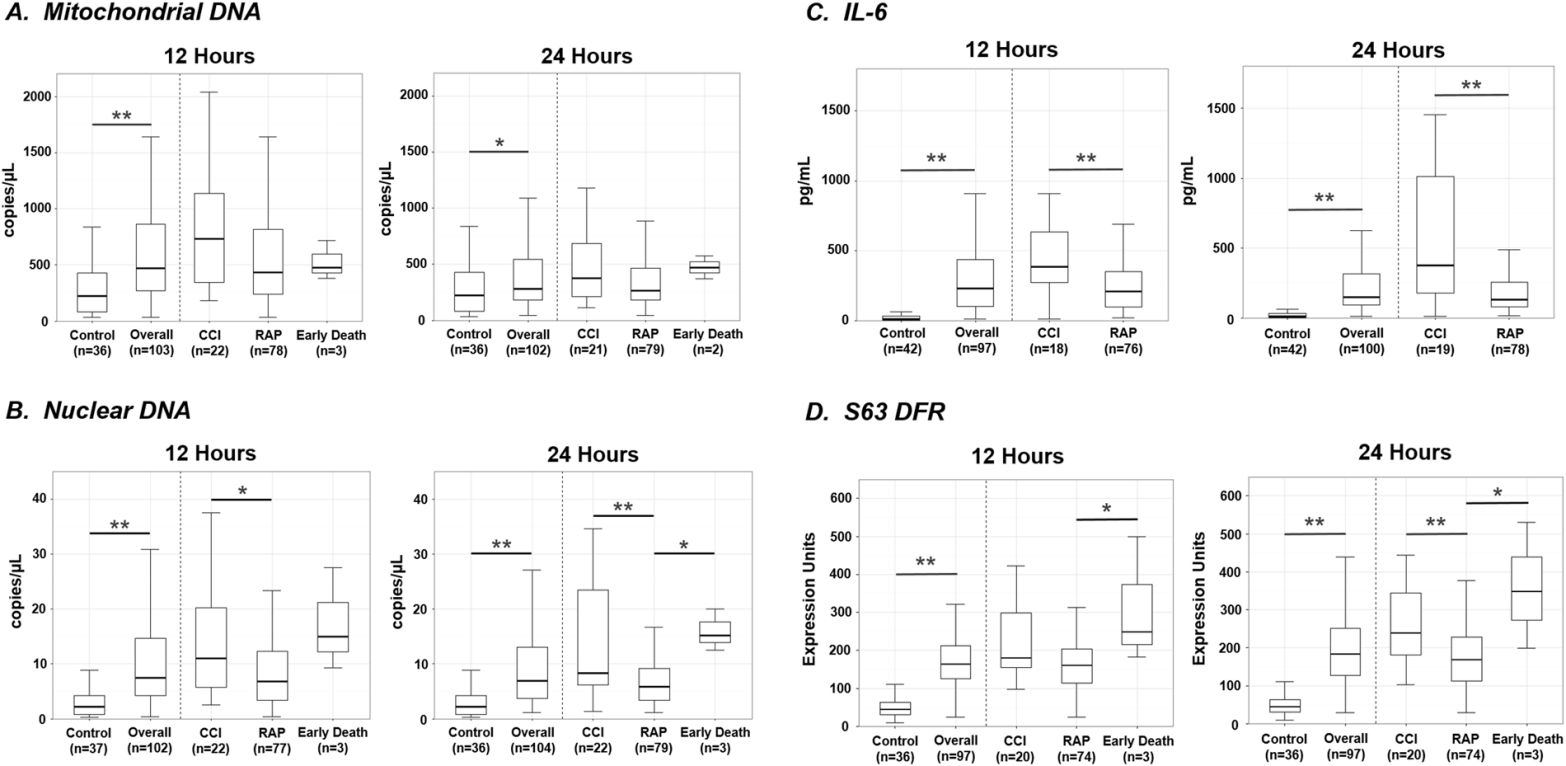
Plasma cfDNA, IL-6 concentrations and blood leukocyte transcriptomics in healthy subjects and blunt trauma patients at ≤12 and 24 hours after injury. Panel A. Cell-free mtDNA copy number at ≤12 and 24 hours. Values represent medians, quartiles (box plots) and 95%iles (whiskers). On the left side are healthy controls and the total cohort of trauma patients in whom values were obtained. The right side contains the trauma cohort broken into subjects who died during hospitalization, rapidly recovered (RAP) or developed chronic critical illness (CCI). *p < 0.05, **p < 0.01. Panel B. Cell-free ncDNA concentrations at 12 and 24 hours. Panel C. Plasma IL-6 concentrations. Median, quartiles for patients who suffered an early death have not been included on the figure because it would expand the y-axis and compress the appearance of the other groups. In the early death patients, the median IL-6 concentration was 1501 pgs/ml (812, 4349) at ≤12 hours and 372 pgs/ml (238, 3822) at 24 hours. Panel D. s63 leukocyte transcriptomics. The expression of 63 genes was reduced to a single metric as described in the Materials and Methods, and previously published^43^.

Mitochondrial cytochrome C oxidase subunit III (*MT-CO3*) and human rhodopsin (*RHO*) copy number were used to represent mtDNA and ncDNA plasma cfDNA concentrations, respectively. Compared to age, gender and race-ethnicity-matched healthy subjects, both mtDNA and ncDNA copy number were significantly elevated in trauma patients at ≤12 and 24 hours (Fig. 1 panels A,B). Similarly, plasma IL-6 concentration, a well-accepted biomarker of the magnitude of the systemic inflammatory response, was also significantly increased as compared to controls at both ≤12 and 24 hours (Fig. 1 panel C)^21^. Although statistical analysis of the transcriptomic response cannot be performed since the metric is derived from the difference between healthy control and the trauma subjects, and therefore is not independent, there was a five-fold increase in the transcriptional metric at both ≤12 and 24 hours (Fig. 1 panel D).

### Differences in early cfDNA and inflammatory responses in trauma patients with alternate clinical trajectories

Since 28-day mortality was low in this cohort (6 of 104, 6%), we used the development of CCI as our primary clinical outcome variable. Unexpectedly, when patients were dichotomized based on their clinical trajectories, differences in mtDNA concentrations were not significantly different at either ≤12 or 24 hours in patients with CCI versus rapid recovery (Fig. 1 panel A). In contrast, ncDNA concentrations were significantly higher at ≤12 and 24 hours in CCI versus rapid recovery patients (Fig. 1 panel B). Similarly, both plasma IL-6 concentrations and the leukocyte transcriptomic metric (S63) were significantly higher in CCI patients at both ≤12 and 24 hours than in those who rapidly recovered (Fig. 1 panel C,D).

### Correlations and ability of cfDNA, IL-6 and transcriptomics to predict clinical trajectory

Since mtDNA has been proposed to serve as an endogenous alarmin responsible for the immediate and early inflammatory responses, including cytokine and genomic *‘storms’*, we initially looked at univariate correlations between cfDNA concentrations and inflammatory and genomic markers (Table 3). We focused on 24 hour measurements, since the correlations obtained within 12 hours post trauma were consistently less than seen at 24 hours. This is likely due to the hemodynamic instability of these patients in the immediate post-trauma period. As anticipated, the strongest correlations among alarmins and inflammatory markers were between the concentrations of ncDNA and mtDNA (*rho* = 0.646, p < 0.001). Surprisingly, mtDNA concentrations were not correlated with either ISS or APACHE II scores, while ncDNA was correlated with injury burden (ISS; *rho* = 0.203, p = 0.040). Both ncDNA and mtDNA correlated with an abnormal leukocyte transcriptomic response (ncDNA; *rho* = 0.219, p = 0.031 and mtDNA; *rho* = 0.214, p = 0.036) but neither were correlated with plasma IL-6 nor maximal lactate concentrations (Table 3). In contrast, the S63 leukocyte transcriptomic metric was most strongly correlated with shock severity, physiologic derangement, and elevated IL-6 levels.

**Table 3:** Correlations Among Biomarkers And Clinical Determinants at 24 Hours^a^.

	ncDNA	mtDNA	WBC	Age	ISS	APACHE II	Initial Lactate	Maximum Lactate^b^	IL-6	S63 DFR
**ncDNA**	1	**0**.**646**	0.149	−0.051	**0**.**203**	0.030	0.166	0.130	0.108	**0**.**219**
p-value		**<0**.**0001**	0.3539	0.6102	**0**.**0397**	0.7642	0.093	0.1891	0.2835	**0**.**0313**
N	104	**104**	41	104	**103**	104	104	104	100	**97**
**mtDNA**		1	0.287	−0.089	0.084	0.040	−0.014	−0.026	0.092	**0**.**214**
p-value			0.0691	0.37	0.399	0.6889	0.8873	0.7956	0.3611	**0**.**0356**
N		104	41	104	103	104	104	104	100	**97**
**WBC**			1	−0.113	−0.008	0.082	−0.057	0.108	−0.056	0.044
p-value				0.4803	0.9605	0.6097	0.7226	0.5016	0.7326	0.8061
N			41	41	40	41	41	41	39	34
**Age**				1	−0.178	0.123	−0.093	−0.007	−0.088	0.067
p-value					0.0721	0.2142	0.3456	0.9402	0.3812	0.5139
N				104	103	104	104	104	100	97
**ISS**					1	**0**.**420**	**0**.**353**	**0**.**339**	**0**.**347**	**0**.**371**
p-value						**<0**.**0001**	**0**.**0003**	**0**.**0005**	**0**.**0004**	**0**.**0002**
N					103	**103**	**103**	**103**	**99**	**97**
**APACHE II**						1	**0**.**280**	**0**.**379**	**0**.**338**	**0**.**440**
p-value							**0**.**0039**	**<0**.**0001**	**0**.**0006**	**<0**.**0001**
N						104	**104**	**104**	**100**	**97**
**Initial Lactate**							1	**0**.**814**	**0**.**208**	**0**.**327**
p-value								**<0**.**0001**	**0**.**0376**	**0**.**0011**
N							104	**104**	**100**	**97**
**Maximum Lactate**								1	**0**.**253**	**0**.**420**
p-value									**0**.**0111**	**<0**.**0001**
N								104	**100**	**97**
**IL-6**									1	**0**.**509**
p-value										**<0**.**0001**
N									100	**93**
**S63 DFR**										1
p-value										
N										97

*Abbreviations*: ncDNA = nuclear DNA; mtDNA = mitochondrial DNA; WBC = white blood cell; ISS = injury severity score; APACHE II = acute physiology and chronic health evaluation II; DFR = distance from reference

^a^Spearman correlation coefficients (*rho*) were used to determine the relationships between variables. Corresponding p-values along with the number of patients upon which these correlations were derived are also listed.

^b^Maximum lactate was the greatest lactate (mmol/L) within 24 hours of injury.

**Figure 2:**
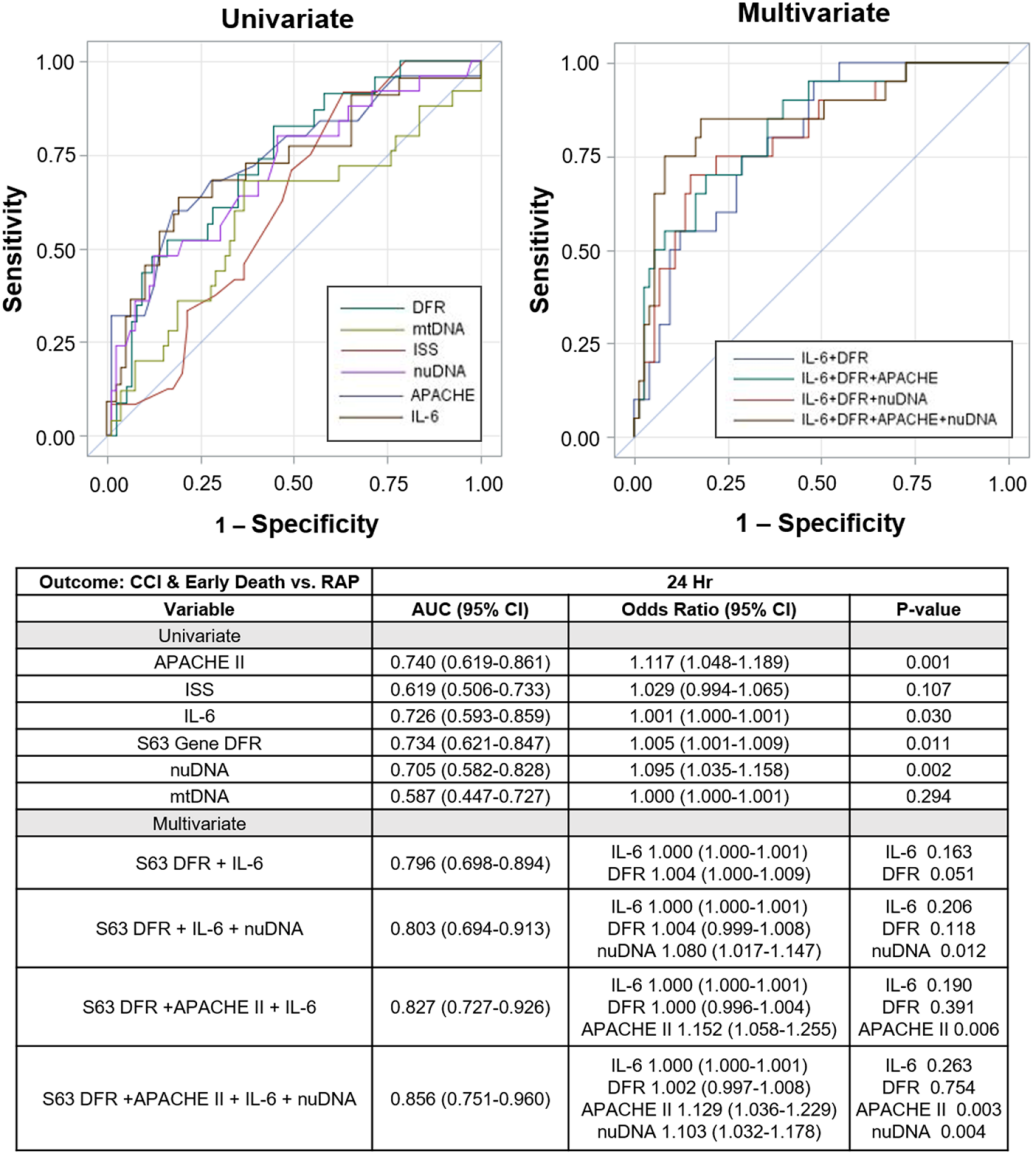
Areas under the receiver operating curves for individual metrics at 24 hours. The predictive ability of cfDNA copy number, plasma IL-6 concentrations and leukocyte transcriptomics (s63 DFR) to predict CCI and a rapid recovery are presented. Values are obtained at 24 hours and compared to APACHE II scores. The accompanying table provides AUCs and relative risks.

Subsequently, the ability of cfDNA concentrations, plasma IL-6 and transcriptomic changes measured at 24 hours after blunt trauma to predict clinical trajectory over 14 days was assessed using area under the receiver-operating curves **(**Fig. 2). The leukocyte transcriptomic response (S63), followed by plasma IL-6 concentrations, and then ncDNA were nearly identical in their predictive ability. In contrast, mtDNA concentrations could not distinguish RAP from CCI at 24 hours (OR 1.00 [95% CI 1.000–1.001]) (Fig. 2).

## Discussion

In this report, we demonstrate that in the immediate 24-hour period following severe blunt trauma, there is a significant increase in the amount of circulating cell-free ncDNA and mtDNA, as measured with a highly quantitative droplet digital PCR (ddPCR) technology. Importantly, ncDNA, but not mtDNA, copy number was significantly greater in patients who subsequently developed CCI versus RAP, and were as predictive of clinical trajectory as IL-6 concentrations and an abnormal leukocyte transcriptomic response.

Previously, authors have suggested that plasma mtDNA concentrations in particular could serve as a diagnostic biomarker for the severity of the injury response^9–14^. In fact, some investigators have recently speculated that removal of circulating mtDNA could be a therapeutic option in this patient population^22^. All of these proposals are based on the well-known properties of mtDNA as endogenous alarmins^3^. Recognized by PRRs, primarily intracellular, mtDNA in particular can activate innate immunity and inflammation through multiple intracellular signaling pathways^23,24^.

Our failure to detect any association between mtDNA copy number and injury severity or clinical outcomes is in contrast to several earlier publications^9–14^. There are two potential explanations. First, we excluded subjects with severe traumatic brain injury and those who died within 48 hours of injury. This eliminated the neurologically impaired or mortally-injured patient secondary to uncontrollable hemorrhage or refractory shock, since our focus was on the association between cfDNA concentrations, the early inflammatory response and long-term clinical trajectories. In this manner, our patients were less lethally injured and more homogenous since we only included immediate survivors with the potential for recovery, but at high risk of a complicated clinical course. Secondly, we used a highly precise ddPCR technology that does not rely on exponential quantitation based on the number of PCR cycles. We purposefully removed any whole cells, whole mitochondrion and microaggregates by repeated high speed centrifugation prior to assay to assure that the mtDNA and ncDNA measured were indeed cell-free, which was not always the case in earlier studies.

We would also conclude that extracellular cfDNA probably does not directly contribute to the systemic inflammatory response; rather, it more likely serves as a biomarker of cellular stress or cell death. Furthermore, the release of cfDNA is more likely to be representative of injury burden secondary to direct tissue damage as opposed to shock severity or early physiologic derangement. Interestingly, ncDNA and mtDNA concentrations were detectable in the plasma of healthy subjects, presumably in the absence of any systemic inflammatory response. The presence of mtDNA and ncDNA in the cell-free plasma of healthy controls has been previously reported, but why it does not elicit inflammation is unclear^9–11^. Unlike ncDNA, mtDNA does not circulate bound to nucleosomes, and is highly charged, binding to a variety of plasma proteins. Cellular receptors recognizing single and double-stranded DNA are primarily located intracellularly on the endoplasmic reticulum, and are therefore physically separated from their plasma ligand^25,26^. Poli *et al*. have argued that plasma cfDNA must bind to IL-26, a member of the IL-10 superfamily, for intracellular transport and binding to intracellular PRRs^27^. Others have speculated that circulating and intracellular mtDNA may preferentially bind to multiple extra- or intracellular proteins including LL37, HMGB1, TFAM and other cationic proteins to signal through either cytosolic RAGE, TLR, STING or cGAS signaling pathways^23,24^. This would imply that the ability of plasma or extracellular cfDNA to serve as an injured tissue alarmin requires the trauma-induced increased production of transporter proteins across the cell membrane or intracellularly.

Plasma ncDNA may better predict clinical trajectories because, in contrast to mtDNA, the presence of ncDNA has been directly attributed to cell death and release of cellular contents^28–30^. The exact cellular source of this ncDNA remains undetermined in trauma patients, although necroptosis and NETosis of blood neutrophils clearly contributes to extracellular DNA^31^. Snyder *et al*. and Ulz *et al*. deep-sequenced plasma cfDNA from healthy control subjects, and based on the nucleosome patterns, concluded that plasma cfDNA likely results from the death of lymphoid and myeloid cells^32,33^. If this is the case, then circulating cell-free ncDNA should be common in both healthy and trauma patients, and is the result of the normal death and/or release from hematopoietic cells. Whether the increase in ncDNA concentrations after trauma in patients with CCI is from increased lymphocyte, macrophage and dendritic cell death or from injured parenchymal tissues is presently unknown. Interestingly, our data shows that ncDNA is most strongly associated with tissue injury burden, specifically ISS, rather than other metabolic stressors such as shock severity or acute physiologic derangement. This would support the hypothesis that CCI is due to increased immune cell death leading to immunosuppression, placing these patients at increased risk for secondary infections^34^. Regardless of its source, this ncDNA is likely not inherently proinflammatory or immunogenic because of its tight binding to nucleosomes^35^.

Unlike ncDNA, which is almost exclusively released upon cell death, mtDNA can be released by both cell death and active secretory processes^36–38^. Mast cells are known to secrete cell-free mtDNA^39^. In addition, whole mitochondria and mitochondrial fragments are often released during necroptosis and NETosis^40,41^. These organelles or cell fragments are then phagocytosed by myeloid cell populations and mtDNA is released intracellularly from endo-lysosomes for binding to intracellular receptors^41^. Therefore, the reduced ability of cell-free mtDNA concentrations to predict the host inflammatory response and clinical trajectories when compared to ncDNA may reflect differences in the origins and release of these cfDNA.

Our study has multiple limitations that require discussion. The first sample for trauma patients was collected within the first 12 hours after injury due to variability in patient presentation at the emergency department following injury. This may introduce significant variation in plasma cytokine and cfDNA concentrations given the variable number of hours between injury and initial sample collection. Additionally, we did not quantify cfDNA levels beyond 24 hours post-injury, limiting our ability to predict how cfDNA concentration changes following the first day. Further work is necessary to determine how cfDNA concentrations change beyond one day after injury, and whether these levels are additionally related to volume resuscitation or major surgical interventions. In addition, our isolation procedure removes cells, mitochondria and microaggregates, it does not remove microparticles, exosomes or ectosomes, and therefore, the source of the cfDNA remains unknown, and how it is affected by blunt trauma.

The findings here both contribute to our understanding of what drives the host inflammatory response, and the utility of various biomarkers of clinical outcome after severe trauma. We found that cfDNA concentrations have only limited association with either the magnitude of the inflammatory response or clinical trajectory, although ncDNA does appear to outperform mtDNA in this regard. The complex host response to severe blunt trauma is likely driven by the release of multiple alarmins simultaneously, and the concentration of any individual alarmin is unlikely to be an effective predictor of clinical outcomes. Therefore, further refinement using a multi-factor biomarker approach is likely necessary to enhance prediction modeling and to improve patient selection for targeted immunotherapies to treat severely-injured trauma patients.

## Materials and Methods

### Study sites and design

This prospective, observational cohort study was conducted over a 3-year period (October 2013 to August 2016) at two United States Level 1 trauma and tertiary care centers: UF Health Hospital, Gainesville, Florida, and Harborview Medical Center, Seattle, Washington. The institutional review board (IRB) of each institution granted approval prior to study initiation. All research was performed in accordance with relevant guidelines/regulations. The study was prospectively registered with *clinicaltrials*.*gov* (NCT01810328), and clinical outcomes previously reported^42^.

### Enrollment and informed consent

Subjects were initially enrolled under a 96-hour waiver of informed consent protocol, which was previously approved and implemented by both institutions. The IRB considered critically injured patients to be a ‘vulnerable’ population, and therefore the purpose of the delayed consent was to permit study patients and their next of kin sufficient time to understand the nature of the study, its risks and benefits. All patients were consented after regaining capacity, or informed consent was provided by the patient proxy in any cases in which patients remained incapacitated.

### Inclusion/exclusion criteria

Inclusion criteria included patients ≥18 years with severe blunt traumatic injury and hemorrhagic shock (systolic blood pressure <90 mm Hg or base deficit of ≥6 meq/L within 60 min of arrival of trauma center). Patients expected to survive ≤48 hours and those with severe traumatic brain injury (Glasgow Coma Scale <8 and abnormal head CT) were excluded. All consecutive patients meeting these criteria in which informed consent was obtained within 96 hours were enrolled.

### Definition of outcomes

Development of CCI was the primary clinical outcome variable. CCI was defined as prolonged ICU admission (≥14 days during index hospitalization) with evidence of persistent organ dysfunction. This definition is based upon prior data that patients meeting these criteria demonstrate a prolonged, dysregulated genomic response to injury, persistent organ dysfunction, and adverse outcomes^2,19,20^. Persistent organ dysfunction was defined using the Modified Marshal Score criteria requiring either greater than or equal to 2 in the renal (serum creatinine ≥1.9 mg/dl [without dialysis]) or pulmonary (PaO_2_/FIO_2_ ≤ 300) categories, or greater than or equal to 1 in the cardiac category (systolic blood pressure <90 mm Hg, or use of vasopressors). MOF was defined as a maximum Denver MOF score ≥3. Patients with an ICU length of stay <14 days without persistent organ dysfunction were classified as RAP. TTR in these trauma patients was defined as the first day meeting organ failure recovery criteria in all organ systems without subsequent days of organ failure (Table 4 outlines organ failure recovery criteria). Finally, early death was defined as death within 7 days of injury in patients surviving greater than 48 hours.

**Table 4:** Parameters for Time to Recovery.

Definition of Time to Recovery (TTR)^a^ First day meeting organ failure recovery criteria in all systems listed below, without any subsequent days with further organ system failure.
Cardiovascular recovery Mean arterial pressure >60 mmHg and no inotropic/vasopressor support (dopamine, dobutamine, epinephrine, norepinephrine, phenylephrine or vasopressin).
Hematologic recovery Platelet count >120,000/µL
Hepatic recovery Serum bilirubin <3 mg/dL
Renal recovery No dialysis and creatinine <1.3 mg/dL
Respiratory recovery No mechanical ventilation or PaO2/FiO2 > 300

^a^A surviving patient who did not recover by day 28 or died prior to day 28 was assigned a TTR value of 30 days. Parameters used to define TTR were established by Cuschieri *et al*.^19^.

### Sample collection and processing

EDTA-anticoagulated blood samples were collected from trauma patients within 12 hours following trauma, and again at 24 hours. A single blood collection was performed on thirty-seven age, gender and race/ethnicity-matched healthy control subjects who provided written informed consent. In order to measure IL-6 and the levels of freely circulating DNA in plasma, samples were centrifuged first at 200 × g and stored at −80 °C. For cfDNA, the plasma was then thrice centrifuged at 5000 × g to remove whole mitochondria, residual leukocytes, and apoptotic vesicles. At this speed, exosomes, microparticles and ectosomes are likely not removed. For transcriptomic measurements, total blood leukocytes were processed and extracted as previously described^43,44^. An aliquot of whole blood was initially centrifuged at 500 × g and the buffy coat layer was removed; residual erythrocytes were then lysed with 15 ml of erythrocyte lysis buffer (Qiagen, Valencia, CA) and the leukocyte-rich fraction was collected by centrifugation^45^. Cells were washed twice with cold lysis buffer, and after the final centrifugation, the dry cell pellet was lysed with RLT buffer (Qiagen, Valnecia, CA), and stored at −80 °C until analysis.

### Analytical methods

The number of copies of a representative mtDNA and ncDNA sequence was quantified using the Bio-Rad QX 200 Droplet Digital PCR (ddPCR) System with EvaGreen™ fluorescent dye (Hercules, California, USA) as previously described^46^. Briefly, primers and probes targeting representative mtDNA and ncDNA were selected for quantification of absolute copies/µL. Enzyme activation followed by denaturation and annealing cycles and enzyme deactivation were performed per the manufacturer’s instructions. At least 50,000 droplets were read and quantified with the number of positive droplets determining the absolute copy number and concentration. Human mitochondrial cytochrome C oxidase subunit III (*MT-CO3*), and rhodopsin *(RHO)* DNA sequences were used to represent mtDNA and ncDNA, respectively. Primer sequences are provided in Supplementary Table 1.

To quantify the transcriptomic response from blood leukocytes, we used a metric comprised of the expression of 63 genes, using the NanoString FLEX™ (Seattle, Washington, USA) platform that has been prospectively validated to predict clinical trajectories in a similar population of severe trauma patients Supplementary Table 2 ^43^. Briefly, leukocyte RNA captured from EDTA-anticoagulated blood samples, was extracted, the NanoString™ platform was performed per the manufacturer’s guidelines, and fold-changes of the 63 genes of interest were calculated and reported as the difference-from-related (DFR) metric. Very briefly, the Nanostring platform uses specific capture probes for the 63 genes (and 7 housekeeping genes) and unique fluorescently labeled probes to quantitate the number of captured transcripts simultaneously. Unlike, qPCR, Nanostring requires no amplification, is linear and has analytical reproducibility less than 5–10% depending upon the quantity of mRNA. Plasma IL-6 concentrations were determined using the Luminex Magpix™ (Austin, Texas, USA) platform according to the manufacturer’s recommendations. Clinical outcomes were prospectively adjudicated by the investigators, and the clinical dataset locked before predictive modeling commenced.

### Statistics

The genomic metric, S63, was calculated as a difference from reference, as previously described^43^. Very briefly, the single metric is derived from the sum of the square root differences in gene expression from age, gender matched healthy controls, adjusted for the individual gene variance, and squared to eliminate positive and negative differences from control.

Due to the high frequency of data failing normality tests, data are presented as medians and quartiles, and are compared using the Kruskal-Wallis test. Categorical variables are presented as frequencies and percentages, and are compared using Fischer’s exact test. For all univariate and multivariate analyses, we report adjusted odds ratios (OR) with 95% confidence intervals (95% CI). Area under the receiver operating curve values (AUC) and Hosmer-Lemeshow goodness-of-fit test were used to assess model discrimination and fit. Spearman correlation coefficients (*rho*) were used to determine the relationship between quantitative variables. All analyses were performed using SAS (v.9.4, Cary, North Carolina, USA).

## Supplementary information

Supplementary Material

## Data Availability

The datasets generated during and/or analyzed during the current study are available from the corresponding author on reasonable request.
