# Supplementary material for: Cell-free nuclear, but not mitochondrial, DNA concentrations correlate with the early host inflammatory response after severe trauma
Source: Sci Rep. 2019 Sep 20;9:13648. doi: 10.1038/s41598-019-50044-z (PMC6754448; doi:10.1038/s41598-019-50044-z)
Supplement: Supplementary file 1 — Supplementary Material [file 41598_2019_50044_MOESM1_ESM.docx]

**Cell-free nuclear, but not mitochondrial, DNA concentrations correlate with the early host inflammatory response after severe trauma**

Julie A. Stortz, MD^1*^; Russell B. Hawkins, MD^1*^; David C. Holden, PhD^1*^; Steven L. Raymond, MD^1^; Zhongkai Wang, MS^2^; Scott C. Brakenridge, MD^1^; Joseph Cuschieri, MD^3^; Frederick A. Moore, MD^1^; Ronald V. Maier, MD^3^; Lyle L. Moldawer, PhD^1^; and Philip A. Efron, MD^1^

**Author Affiliations:**

*^1^Department of Surgery, University of Florida College of Medicine, Gainesville, FL 32610, USA.*

*^2^Department of Biostatistics, University of Florida College of Medicine, Gainesville, FL 32610, USA.*

*^3^Department of Surgery, University of Washington School of Medicine, Seattle, WA 98104, USA.*

*Authors contributed equally.

**Supplementary Table 1. Primers Utilized to Study Circulating DNA**^a^

| Primer | Sequence 5’🡪3’ | Template Strand | Length | Start | Stop | Tm | GC% | Self Complementarity | Self 3’ Complementarity | |
| --- | --- | --- | --- | --- | --- | --- | --- | --- | --- | --- |
| Human Rhodopsin Forward Primer | CCTTCTCCAATGCGACGGGT | Plus | 20 | 329 | 348 | 62.52 | 60.00 | 3.00 | 2.00 |  |
| Human Rhodopsin Reverse Primer | GGTGCTGGTGAAGCCACCTAG | Minus | 21 | 576 | 556 | 62.99 | 61.90 | 4.00 | 4.00 |  |
| *Rhodopsin product length: 248 base pairs | | | | | | | | | |  |
|  | | | | | | | | | |  |
| Mitochondrial Cytochrome C Subunit III Forward Primer | ATGACCCACCAATCACATGC | Plus | 20 | 9207 | 9226 | 58.52 | 50.00 | 4.00 | 2.00 |  |
| Mitochondrial Cytochrome C Subunit III Reverse Primer | ATCACATGGCTAGGCCGGAG | Minus | 20 | 9309 | 9290 | 61.76 | 60.00 | 4.00 | 2.00 |  |
| *Mitochondrial cytochrome C subunit III product length: 103 base pairs | | | | | | | | | |  |

^a^ Primers were designed to measure circulating human rhodopsin (ncDNA) and mitochondrial cytochrome C subunit III (mtDNA).

**Supplementary Table 2. Genes Selected in S63 Metric^a^ and Subgroup Classification**

| **Subgroup Classification** | **HUGO Gene Name** | **Abbreviated Function** |
| --- | --- | --- |
| Adaptive Immunity | CD74 | MHCII cofactor |
| Adaptive Immunity | GNLY | cytotoxic granules of T cells |
| Adaptive Immunity | HLADMA | class II alpha |
| Adaptive Immunity | HLADMB | class II beta |
| Adaptive Immunity | HLADPA1 | class II alpha |
| Adaptive Immunity | HLADPB1 | class II beta |
| Adaptive Immunity | HLADQA1 | class II alpha |
| Adaptive Immunity | HLADQB1 | class II beta |
| Adaptive Immunity | HLADRA | class II alpha |
| Adaptive Immunity | HLADRB1 | class II beta |
| Adaptive Immunity | IFI44 | antiviral |
| Adaptive Immunity | IFI44L | antiviral |
| Adaptive Immunity | IFI6 | regulation of immune cell apoptosis |
| Adaptive Immunity | IFIT1 | inhibits viral replication |
| Adaptive Immunity | IFIT2 | inhibits viral replication |
| Adaptive Immunity | IFIT3 | inhibits viral replication |
| Adaptive Immunity | IFIT5 | myeloid cell differentiation |
| Adaptive Immunity | LCN2 | NGAL, inhibits bacterial activity |
| Adaptive Immunity | MX1 | antiviral protein |
| Adaptive Immunity | NAIP | inhibitor of apoptosis |
| Adaptive Immunity | OAS1 | inhibitor of viral replication |
| Adaptive Immunity | OAS2 | inhibitor of viral replication |
| Adaptive Immunity | OAS3 | viral infection inhibition |
| Adaptive Immunity | PMAIP1 | regulation of lymphocyte apoptosis |
| Adaptive Immunity | RSAD2 | interferon signaling |
| Adaptive Immunity | VNN1 | T cell migration |
| Adaptive Immunity | XAF1 | inhibitor of IAP (inhibitor of apoptosis protein) |
| Endothelial Biology | CEACAM6 | cell adhesion |
| Endothelial Biology | HERC5 | endothelial cell ubiquination |
| Endothelial Biology | HGF | endothelial cell growth |
| Endothelial Biology | LRG1 | cell adhesion, PMN maturation |
| Endothelial Biology | PDGFC | angiogenic, proliferative responses |
| Endothelial Biology | TGFBI | inhibits cell adhesion |
| Endothelial Biology | THBS1 | endothelial protein binding fibrinogen |
| Inflammatory Response | ANKRD55 | CD4 and CD14 nuclear protein, inflammation |
| Inflammatory Response | CD24 | PMN and B cell growth factor |
| Inflammatory Response | CEACAM8 | CD66b |
| Inflammatory Response | EPSTI1 | NF-kB activation |
| Inflammatory Response | IL1R1 | inflammatory gene receptor |
| Inflammatory Response | IL1R2 | inflammatory gene receptor |
| Inflammatory Response | ISG15 | chemotactic to PMNs, and antiviral |
| Inflammatory Response | LTF | PMN granular protein, antibacterial |
| Inflammatory Response | MMP8 | PMN collagenase |
| Inflammatory Response | OLFM4 | anti-apoptotic, cell adhesion,  inflammation |
| Inflammatory Response | PTGS2 | prostaglandin biosynthesis |
| Inflammatory Response | TCN1 | neutrophil granules |
| Other | AGFG1 | nuclear transport protein |
| Other | ATP6V1C1 | vacuolar ATPase |
| Other | CDK5RAP2 | cyclin dependent kinase |
| Other | CMPK2 | nucleotide synthesis |
| Other | DACH1 | DNA transcription factor |
| Other | FLJ39051 | unknown |
| Other | FOLR3 | folate receptor |
| Other | GALNT14 | Golgi complex transporter |
| Other | GRB10 | kinase accessory proteins |
| Other | LOC100127983 | uncharacterized |
| Other | MIAT | nuclear matrix |
| Other | NSUN7 | sperm motility |
| Other | OLAH | fatty acid biosynthesis |
| Other | PCOLCE2 | apolipoprotein catalyst |
| Other | SIPA1L2 | cell regulation |
| Other | SLC26A8 | ion transport in spermocytes |
| Other | TDRD9 | DNA methylation in gametes |

^a^ Derivation of Genomic Metric: A single metric was derived from the difference in expression values for 63 genes (difference from reference; DFR) involved in inflammation, activation of endothelium, and the adaptive immune response from age, race/ethnicity, and gender matched healthy subjects using the equation: S63 = ∑*_probe_* *_sets_* (*e_i_*−*M_i_*)^2^*/V_i_* where e_i_ is the patient’s expression level for probe set *i*, and M_i_ and V_i_ are the appropriate sepsis group mean and variance for the *i*^th^ probe set.
